# Glioblastoma and Immune Checkpoint Inhibitors: A Glance at Available Treatment Options and Future Directions

**DOI:** 10.3390/ijms251910765

**Published:** 2024-10-07

**Authors:** Silvia Mara Baez Rodriguez, Ligia Gabriela Tataranu, Amira Kamel, Serban Turliuc, Radu Eugen Rizea, Anica Dricu

**Affiliations:** 1 Neurosurgical Department, Bagdasar-Arseni Clinical Emergency Hospital, 041915 Bucharest, Romania; mara.silvia@icloud.com (S.M.B.R.); kamel.amyra@yahoo.com (A.K.); rizea.radu.eugen@gmail.com (R.E.R.); 2 Neurosurgical Department, Carol Davila University of Medicine and Pharmacy, 020022 Bucharest, Romania; 3 Medical Department, University of Medicine and Pharmacy “G. T. Popa”, 700115 Iasi, Romania; serban_turliuc@yahoo.com; 4 Biochemistry Department, Carol Davila University of Medicine and Pharmacy, 020022 Bucharest, Romania; anica.dricu@live.co.uk

**Keywords:** glioblastoma, immune checkpoint inhibitors, immunosuppression, blood-brain barrier, tumor microenvironment

## Abstract

Glioblastoma is known to be one of the most aggressive and fatal human cancers, with a poor prognosis and resistance to standard treatments. In the last few years, many solid tumor treatments have been revolutionized with the help of immunotherapy. However, this type of treatment has failed to improve the results in glioblastoma patients. Effective immunotherapeutic strategies may be developed after understanding how glioblastoma achieves tumor-mediated immune suppression in both local and systemic landscapes. Biomarkers may help identify patients most likely to benefit from this type of treatment. In this review, we discuss the use of immunotherapy in glioblastoma, with an emphasis on immune checkpoint inhibitors and the factors that influence clinical response. A Pubmed data search was performed for all existing information regarding immune checkpoint inhibitors used for the treatment of glioblastoma. All data evaluating the ongoing clinical trials involving the use of ICIs either as monotherapy or in combination with other drugs was compiled and analyzed.

## 1. Introduction

Glioblastoma (GBM) is the most common and aggressive of all brain tumors, accounting for about 14.2% of brain tumors [1,2]. It is a rapidly growing tumor that develops spontaneously within the brain from various glial cell types and is highly infiltrative in the adjacent brain tissue [3]. The median overall survival (OS) after diagnosis is between 14.6 and 20.5 months, with older patients having a poorer prognosis, with an average survival of less than 8.5 months [4,5,6]. Less than 5% have a five-year or more OS [6].

Hence, it is imperative to improve the current treatment options for GBM [7]. The current standard of care (SOC) for newly diagnosed GBM patients involves maximal surgical resection to reduce the bulk mass of the tumor, followed by radiotherapy and concomitant and adjuvant chemotherapy with temozolomide (TMZ) [8]. At a microscopic level, GBMs invade the brain beyond the tumor’s gross radiographic margins, making it impossible to perform a complete surgical resection. According to 2021 EANO guidelines, the extent of the surgical resection should be assessed postoperatively using an MRI scan in the first 24 to 48 h post-procedure [9]. The tumor fragments harvested either by surgery or biopsy are used to make a histological and molecular diagnosis. Patients with tumors harboring the methylated MGMT promoter are most likely to benefit from this treatment scheme, but these are only a third of the cases. TMZ promotes base methylations, resulting in tumor cell death in the absence of an effective DNA damage repair system.

Nowadays, many clinical trials are trying to find new treatments capable of prolonging the life expectancy of GBM patients. One of the treatments approved by the FDA, with good results shown in a phase 3 clinical trial, is the use of TTFs—tumor treating fields [10], represented by low-intensity alternating electric fields delivered to the scalp of GBM patients, inducing tumor cell mitosis [10,11,12]. High cost, skin toxicity, and patient compliance are a few of the reasons TTFs were not introduced in the GBM SOC protocol [13,14].

The 2021 guidelines released by the World Health Organization classify GBMs as 4th-grade adult-type diffuse glioma according to their molecular and histopathological characteristics. Based on their molecular features GBMs are characterized by their isocitrate dehydrogenase (IDH) wild-type status, retained expression of nuclear Alpha thalassemia/mental retardation X-linked syndrome (ATRX), intact chromosome arms 1p and 19q, and absence of mutations in histone H3 genes. From a histological point of view, GBM presents with microvascular proliferation and necrosis and key molecular alteration such as the mutation of the telomerase reverse transcriptase (TERT) promoter, the amplification of EGFR (epidermal growth factor receptor), and the combined gain of the entire chromosome 7 and loss of whole chromosome 10 [1].

GBM is a highly recurrent tumor, with median OS at recurrence being from 2 to 9 months and progression-free survival (PFS) from 1.5 to 6 months [15,16,17]. Treatment options for recurrent GBM are limited and include secondary resection, when possible, chemo-radiotherapy, and experimental treatments.

Antibodies are potent therapeutic tools in treating various cancers starting from the late 1980s [18,19]. Nowadays, the use of immunotherapy in managing several previously intractable tumors has brought remarkable results. It seems that glioblastomas’ capacity to evade the immune system results in poor outcomes after using immunotherapeutic approaches. It is important to understand the specific mechanisms of immunosuppression in glioblastoma, with the goal of finding ways in which immunotherapy could be used against this disastrous disease.

## 2. Obstacles Implicated in the Therapeutic Failure of Immunotherapy

The difficult task of developing new treatment options for GBM is influenced by numerous characteristics that are most likely responsible for its poor prognosis. Some of these are represented by the challenging anatomical location, the tumor invasiveness with diffuse patterns of growth and infiltrating characteristics, the intra- and intertumoral heterogeneity, the blood-brain barrier (BBB), and the immunosuppressive nature of the tumor microenvironment (TME) [20,21] including dendritic cells, monocytes, CD4+ Tcell, CD8+ Tcell, tumor-associated neutrophils (TANs), tumor-associated macrophages (TAMs), microglia, myeloid-derived suppressor cells (MDSCs).

The brain is vital in regulating the fundamental processes of our body and mind. Surgical resection is possible only when the tumor’s location is within non-eloquent areas of the brain that don’t affect movement, speech, vision, or memory. The patient’s quality of life needs to be prioritized over the extent of resection, preventing permanent neurological deficits. Today, there are multiple tools and different techniques available for surgeons to avoid any undesirable events, such as pre-operative techniques represented by functional MRI imaging, navigated transcranial magnetic stimulation, magnetoencephalography, and diffusion tensor imaging, and intra-operative techniques—ultrasonography, electrostimulation, cerebral perfusion measurements, and 5-ALA (aminolaevulinic) tumor labeling [22,23].

GBM cells are associated with a high invasive capacity, leading to treatment resistance, recurrence, and poor OS. The extracellular matrix (ECM) is modified and degraded by the tumoral cells, causing an invasive behavior through glutamate release and Ca^2+^ signaling pathways [24]. The GBM core cells are more proliferative, while the cells located at the tumor periphery are more invasive, moving as individual cells [25] or in groups [26,27], penetrating the surrounding normal brain tissue, migrating along brain parenchyma, white matter tracts, blood vessels, and subarachnoid spaces [28,29], while remodeling the extracellular matrix and their cytoskeleton and energy metabolism [29,30,31]. The invasiveness of GBM cells makes complete surgical resection impossible, with the remaining tumoral cells causing rapid tumor recurrence within months after initial surgery [32].

GBM presents a high heterogeneity, both intratumoral (within various parts of the same tumor) and intertumoral (among different tumors with similar histological characteristics). This causes different responses to treatment based on their molecular profiles. P53-mediated pathway, retinoblastoma protein (RB) pathway, and phosphoinositide 3-kinase (PI3K) pathway are only some cell signaling pathways that can be dysregulated in GBM. Three molecular subtypes of GBMs were identified by The Cancer Genome Atlas, based on molecular analysis: proneural, mesenchymal, and classical [33,34]. Evidence of the presence of various subtypes within the same tumor was found, indicating intra- and intertumoral heterogeneity, with individual cell variations in the gene expression patterns [33,35,36,37]. The classification is meant to help researchers and clinicians discover and deliver more personalized treatment options to patients [38]. It may also help to implement customized immunotherapy approaches [39].

The GBM tumoral cells that comprise the tumoral mass are very different at the epigenetic, transcriptomic, protein, and metabolic levels [40,41]. The therapeutic approaches contribute to phenotypic heterogeneity by modifying the tumor landscape [42], providing survival skills to the tumoral cells with the rapid emergence of cell clones resistant to treatment.

### 2.1. The Blood-Brain-Barrier

The blood-brain barrier (BBB) is a highly selective semipermeable structure that protects the brain from all potentially harmful blood-borne agents and exogenous compounds (drugs, neurotoxins) that may damage the CNS [43,44]. It helps control the circulation of cells, molecules, and ions to and from the blood in order to ensure the proper function of the neurons [45]. The brain is isolated from the bloodstream with the help of tight junctions of the endothelial cells that form intraparenchymal capillaries. They are surrounded by pericytes and astrocytic endfeet [43,46]. The BBB limits the penetration of systemically administered chemotherapeutic agents. The barrier also poses a significant challenge for the delivery of antibody-based therapeutic agents [47], with monoclonal antibodies being considered too large to penetrate the BBB [48]. The BBB makes treating GBM much harder than treating other solid tumors [49].

It is presumed that the expression of the neonatal Fc receptor (FcRn) in the capillary endothelium of the BBB contributes to preventing the delivery of antibodies to the brain parenchyma. FcRn is supposed to cause reverse transcytosis of IgG antibodies from the brain to the blood [50,51]. The distribution of the antibodies to the brain may be improved by preventing the interaction between the FcRn and the Fc antibody domain [50,52].

Due to aberrant neovasculature and irregular blood flow, GBM develops a so-called blood-brain tumor barrier (BTB), which can decrease the results of the treatment when medication is administered systemically and prevents the medication from leaving circulation [49]. The brain-tumor-barrier (BTB) appears at the level of the tumoral core, where the BBB is partially disrupted, causing increased permeability, VEGF overexpression with increased angiogenesis in the hypoxic zones, and the release of cytokines and chemical mediators that lead to the formation of new immature and permeable vessels within the tumor [53,54,55,56,57]. This increased permeability of the BBB may help deliver drugs to the tumor core but fails to offer a solution to the penetration of drugs in the peripherical parts where the invasive cells are located.

Scientists are working to find new ways to increase drug penetration into the brain either by modulating the BBB (modulation of efflux pumps, tight junctions, or the use of receptor agonists) or by enhancing drug liposolubility (using liposomes) [58]. Some approaches have been developed to bypass the BBB:

(1) Using treatments that are better at crossing the BBB’s endothelial cells by decreasing the ability to create hydrogen bonds, polarity, or lipophilicity [59];
(2) Using a monoclonal antibody as a carrier directed against one of the BBB’s transcytosis receptors. This way helps the chemical, which usually is incapable of entering the brain, to be undetected.
(3) With the help of nanotechnology, by using as a carrier a liposome containing an antibody that targets transferrin [60]. Inorganic nanoparticles (IONPs) can also be used to deliver drugs with an iron oxide core, serving at the same time as an imaging agent for MRI, helping to track the delivery of the therapeutic agents to the tumor [60]. Certain peptides can be used, combining them with therapeutic molecules and delivering them to the tumor, protecting the rest of the brain from damage [61].
(4) Some other ways that can be used to allow medicines to enter the brain are radiation, electroporation, and low-intensity ultrasound (LIPU) [62]. LIPU was used in a Phase I/IIa clinical trial in which patients had the SonoCloud-1 device implanted at the skull level to administer pulse sonication. The results showed that LIPU was well tolerated, and carboplatin penetrated the brain after being sonicated [63]. Irreversible electroporation (IRE) was studied in a canine model and proved to break the BBB and eradicate tumor cells [64]. Convection-enhanced delivery (CED) is able to avoid the BBB and deliver medication directly to the tumor or the surrounding area.

The development of therapies that can either pass through, disrupt, or bypass the BBB will improve the effectiveness of ICIs in the future.

### 2.2. Systemic and Local Immunosuppression

The brain presents unique immune characteristics compared with other body parts. In normal conditions, the brain is immunologically quiescent, with the infiltration of circulating immune cells being limited by the BBB [65]. The neural environment is supervised by embryonically derived resident microglial cells that serve as resident macrophages [66], with further immunosurveillance being provided by a specialized lymphatic drainage system [67]. This specialized lymphatic drainage system drains CSF (cerebrospinal fluid) along with immune cells and solutes into deep cervical lymph nodes [67,68]. In inflammatory conditions that increase the permeability of the BBB, APCs (antigen-presenting cells), not usually present in normal parenchymal tissue, rapidly migrate towards it from the adjacent vascular-rich tissues, like choroid plexus and meninges [69], through afferent lymphatics or endothelial venules looking for antigens [69]. Afterwards, they reach the deep cervical lymph nodes, where they can display brain-derived antigens and prime T and B lymphocytes, initiating adaptive immune responses [67,70]. T cells are also present in healthy individuals’ brain parenchyma and CSF [71]. All these characteristics translate to a unique tumor microenvironment.

Studies have shown that GBM patients suffer from pronounced immunosuppression, which affects their overall immune system (systemic) and the immune responses within the tumor environment (local) [72]. Compared with healthy individuals, GBM patients present smaller secondary lymphoid organs, lower MHC—II (major histocompatibility complex class II) expression levels in peripheral blood monocytes, and T-cell lymphopenia [73,74,75]. The consequences of the thymic reduction in size and function are decreased T-cell production, followed by reduced T-cell availability for anti-GBM immunity [76]. The loss of surface sphingosine-1-phosphate receptor (S1P1), which is in charge of the migration of T cells from the thymus and secondary lymphoid organs, causes the confinement of the majority of T cells in the bone marrow (BM) and the lack of migration into the bloodstream [75]. Studies have shown that the immunosuppressive treatment with corticosteroids and TMZ and the circulating cytokines produced by the tumors can support the systemic immunosuppression observed in GBM patients [73,77].

The local immunosuppression appears due to the disruption of the BBB with increased permeability, allowing a great influx of immune cells [78,79]. The glioblastoma tumor microenvironment (TME) is embodied of various components: tumor cells, signaling molecules, vasculature, extracellular matrix, brain resident non-immune cells like neurons and astrocytes, and 20% to 40% lymphoid and myeloid immune cells and bone-marrow-derived macrophages from the circulation [80]. Studies have shown that the concentration of monocyte-derived macrophages (MDMs) and lymphocytes is higher in IDH-wild-type tumors. Although the immune responses should be able to eliminate cancer cells or stop their growth, GBM cells can develop multiple mechanisms to evade immune surveillance and transform the TME, allowing tumor progression. GBM cells communicate with the TME by cell-to-cell contact, soluble molecules, and extracellular vesicles [81,82].

Soluble molecules are secreted by various cells that exist in the TME. They are represented by multiple growth factors and cytokines. There are two categories of molecules:

(1) Tumor-promoting cytokines: includes interleukin (IL-1)3 and basic fibroblast growth factor (bFGF). These promote tumorigenesis.
(2) Immunosuppressive chemical mediators: TGF-β, IL-10, IL-6, and prostaglandin E-2 (PGE-2) [83,84], which are responsible for shifting the immune response from an inflammatory one to a pro-tumoral and wound-healing one. This mutation leads to the decreased capacity of the immune cells to efficiently eliminate tumor cells.

In the GBM TME, there is also a high concentration of CC Chemokine Ligand 2 (CCL2), a very powerful chemoattractant that plays an important role in the recruitment of regulatory T cells (Tregs) and myeloid cells [85].

The GBM extracellular matrix (ECM) composition is modified by the overexpression and increased secretion of laminin, fibronectin, and collagen, resulting in high density and tumor stiffness [86]. These alterations limit chemotherapeutic drugs’ ability to penetrate the tumor, reducing effectiveness. The elevated levels of fibronectin and hyaluronic acid, together with the degradation of ECM caused by metalloproteinases, increase the mobility and invasiveness of glioma cells [87].

The GBM TME is characterized by abnormal vasculature, with poor blood flow at the level of the tumoral core, causing a decrease in oxygenation [88], increasing the expression of hypoxia-inducible factor 1-α (HIF1-α) and promoting angiogenesis and tumor cell invasion [88]. HIF1-α is responsible for the upregulation of immunomodulatory surface ligands like cytotoxic T-lymphocyte-associated protein 4 (CTLA-4) and programmed death ligand 1 (PD-L1), causing the inhibition of the anti-tumor immune responses [89].

Astrocytes normally secrete growth factors and cytokines when the CNS is injured, facilitating tissue repair by astrogliosis [90]. In GBM, this process causes tumor growth. The TME supports the crosstalk between astrocytes and the surrounding microglia. This process activates the JAK/STAT and PD-L1 pathways in the astrocytes, causing an elevated production of anti-inflammatory cytokines like IL-10, TGF-β, and STAT3, creating an immunosuppressive environment [91]. The neurons help facilitate GBM progression by upregulating neuroligin-3, activating the PI3K signaling pathway, and promoting proliferative activity of glioma cells [92].

The main infiltrating populations in GBM are the tumor-associated microglia and tumor-associated macrophages (TAMs). They are attracted toward the tumor due to the high concentration of chemo-attractants, like CCL2, that are secreted by glioma cells [93,94,95]. In the tumor microenvironment, TAMs adopt immunosuppressive and tumor-supportive phenotypes [96]. The increased STAT3 phosphorylation and the suppression of the NF-κB pathway, both caused by the activation of the mTOR signaling pathway, lead to the upregulation of anti-inflammatory cytokines like IL-6 and IL-10 [97]. A decreased expression of surface MHC class II molecules and costimulatory molecules (CD40, CD80, and CD86) is exhibited by TAMs, impairing antigen presentation and the activation of T cells [98,99,100].

GBM overexpresses immune inhibitory proteins, such as ICAM1, which interacts with LFA-1, causing myeloid-derived suppressor cell (MDSC) accumulation within the TME [101]. The immune system is suppressed by MDSCs through multiple mechanisms, including the expression of anti-inflammatory molecules like TGF-β and arginase [102]. The arginase causes the reduction of L-arginine levels that are necessary for TCR expression and function. MDSCs also express PD-L1, which promotes T-cell exhaustion [103,104]. The secretion of nitric oxide and reactive oxygen species is responsible for inhibiting T-cell activity. TMZ can reduce the number of MDSCs. Studies have shown that the MDSC origin and function can vary in GBM by the sex of the patients [105].

Tumor-infiltrating lymphocytes (TILs) can often be dysfunctional and exhausted because of the factors released by the glioma and microenvironmental cells, which include TGF-β, IL-10, and CCL2, causing the recruitment of Tregs (regulatory T cells), MDSCs, and TAMs to the tumor site [106]. TGF-β causes CD4+ cells to upregulate FoxP3 and differentiate into Tregs. These are 25% of TILs associated with poor prognosis in GBM [107]. Tregs contribute to the transition of T cells into regulatory ones, having an immunosuppressive function over natural killer (NK) and CD8+ T cells, helping to generate MDSCs and reduce the antigen presentation capacity of dendritic cells (DCs) [108]. TGF-β1 causes the reduction in the expression of the activating receptor natural killer group 2 (NKG2D) on the CD8+ and NK cell surfaces, inhibiting their cytotoxic effects on GBM cells [109]. Tregs also have the capacity to highly express immune checkpoint molecules like PD-1 and CTLA-4 that suppress their effector functions through interactions with their receptors found on the surface of T cells [110]. Glioma cells suppress lymphocyte activity through various molecules like FasL, PD-L1, PD-L2, CD70, and ganglioside [111,112,113]. The insufficiency of TILs and the accumulation of exhausted T cells in TEM add to the immunotherapy resistance and relapse.

## 3. Immune Checkpoint Therapy

Immunotherapy is bringing a new era in oncology, targeting the reactivation of the immune system’s cell reactions against tumors. These types of approaches have had very good clinical results and, in some cases, full remission of solid tumors, becoming part of the standard of care in various types of cancers [114]. Immune-based treatments have a different impact on each cancer type, depending on tumor intrinsic features and level of immunosuppression. Current investigations regarding GBM include immune checkpoint inhibitors (ICIs), adoptive T-cell therapies, vaccination approaches, and virus-based therapies.

Immune checkpoints are regulatory inhibitory pathways involved in maintaining immunologic homeostasis by modulating the intensity and duration of the immune response. Some of the functions performed by the immune checkpoints include preventing autoimmunity, maintaining self-tolerance in physiologic conditions, and protecting tissues from damage during infection [115]. Cancer usually hijacks such pathways, evading immune system surveillance. The development of immune checkpoint inhibitors was meant to resolve this problem.

Immune checkpoint inhibitors are monoclonal antibodies (mAbs) that target inhibitory checkpoint molecules expressed by the cell membrane of the antigen-presenting cells (APCs) and CD4+ T-cells [116,117]. ICIs have revolutionized cancer treatment by increasing the immune system’s capacity to destroy cancer cells by activating and preventing T-cell exhaustion. ICIs are not implicated in tumor identification but in targeting the tumor-immune cell interface. They are responsible for blocking the cancer cells’ signals used to evade immune responses and allowing immune cells to attack tumors. The effector T cells usually become “exhausted” after prolonged antigenic exposure or tumor-T cell interaction, losing their tumor reactivity. This is known as a hypo-responsive state, characterized by elevated levels of co-inhibitory molecules, immune checkpoints, decreased cytotoxicity, and reduced proliferation capacity [118]. In recent years, various immune checkpoints have been studied, including programmed cell death protein 1 (PD-1) and its ligand PD-L1, CTLA-4, lymphocyte activation gene-3 (LAG-3), T-cell immunoreceptor with immunoglobulin and ITIM domain (TIGIT), T-cell immunoglobulin and mucin domain 3 (TIM-3), V-domain Ig suppressor of T-cell activation (VISTA), and indoleamine 2,3-dioxygenase (IDO).

A large variety of drugs, like ipilimumab, nivolumab, pembrolizumab, atezolizumab, durvalumab, and other anti-PD-1 and PD-L1 inhibitors, are already approved by the FDA for the use in many types of cancers, such as colorectal cancer, gastric cancer, hepatocellular carcinoma, melanoma, classic Hodgkin’s lymphoma, and non-small cell lung carcinoma [119,120,121,122,123]. Although successful in all of these cancer types, they have failed to provide good outcomes in clinical trials for glioblastoma so far.

Glioblastoma and other gliomas are highly attractive targets for immune checkpoint blockade [124]. GBM promotes immunosuppression and is able to escape the immune system through multiple mechanisms. These can be systemic, such as decreased T-cell responsiveness, increased Tregs, decreased monocyte and dendritic cell function, lower level of immunoglobulins, frequent use of corticosteroids, and lymphopenia caused by treatments, or local, such as downregulation of MHC molecules, secretion of TGF-β, VEGF, PG-E2, IL-10, LLT-1, polarization of microglia and tumor-associated macrophages towards the immunosuppressive M2 phenotype [125], decreased T-cell function due to hypoxia, T-cell apoptosis through Fas, infiltration with Tregs, and increased expression of immune checkpoints [126]. Immune checkpoints oversee the regulation of the immune system, exploited by the TME to suppress immune responses towards GBM cells.

Both PD-1 and CTLA-4 are recognized as essential regulators of the balance between efficient T-lymphocyte activation and over-activation of T-cell functions, which may result in damaged immunopathology [127]. The use of ICIs in treating glioblastoma may be influenced by several factors, like the immune environment of the CNS, the presence and differentiation status of TILs, and the potential for interdependent combination therapies. The use of ICIs to block PD-1/PD-L1 and CTLA-4/CD80/86 signaling pathways enhances the efficient immune responses against cancer cells, revitalizing tumor antigen recognition and causing tumor death [128].

**Figure 1:**
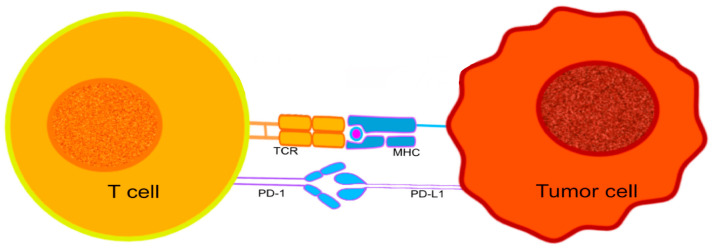
The interaction between PD-1 expressed on the surface of T cells, and PD-L1 expressed on the surface of tumor cells. The immunological checkpoint prevents T-cell activation.

PD-1, a protein that belongs to the immunoglobulin superfamily [129,130], can be expressed by activated T cells, B cells, NK (natural killer), and some monocytes [129,130]. It is particularly important in downregulating the immune system and promoting self-tolerance by suppressing T-cell inflammatory activity [131]. PD-1 is responsible for the release of a series of downstream signals by engaging with one of its ligands, either programmed cell death-ligand 1 (PD-L1) or 2 (PD-L2), leading to the inhibition of cytotoxic T lymphocytes (CTL) [132] (Figure 1). The signals decrease the proliferation of T cells in lymph nodes and their actions in peripheral tissues. The interruption of CTL activities can have positive and negative outcomes on the host’s immunological surveillance mechanisms. The control over CTL activity may help to reduce the possibility of autoimmunity against host antigens, while suppressing CTL activation will contribute to tumor progression, helping the developing tumor cells to evade the host’s immune surveillance [133].

**Figure 2:**
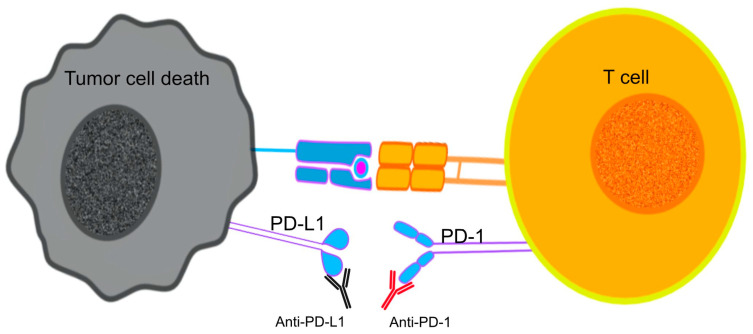
Immune checkpoint inhibitors (ICIs) target T cell exhaustion by blocking immune checkpoints PD-1 and PD-L1, restoring T cell function and antitumor activity.

The use of ICI mAbs to prevent the interaction between PD-1 and its ligands on the surface of T cells contributed to the inhibition of neoplastic growth by reviving the cytotoxic functions of CTLs against tumor antigens, a process caused by the PD-1/PD-L1 induced immunosuppression [134] (Figure 2). The use of PD-1 blockade on PD-expressing T cells was linked to reversed T-cell exhaustion and improved cytokine production [135].

CTLA-4, another strategic immune checkpoint receptor, is mostly expressed on the surface of T cells. It has constitutional similarities with the T-cell co-stimulatory protein CD28, both being able to bind to CD80 and CD86 on APCs. The interaction between CD28 and its ligand CD80 or CD86, along with the binding of T-cell receptor (TCR) to MHC1 (major histocompatibility complex 1), stimulates T-cell activation and proliferation [136]. It seems that there is a preferential binding of CTLA-4 to CD80 or CD86, outcompeting CD28 and therefore interrupting T-cell activation, thus mediating immune evasion and escape mechanisms of tumor cells [137]. CTLA-4 signaling in regulatory T cells (Tregs) contributes to peripheral tolerance by promoting their suppressive function.

Scientists are looking for potential biomarkers associated with ICI responsiveness in GBM patients [138]. Some of these are associated with the differentiation status of tumor-infiltrating lymphocytes and the function of cytotoxic CD8+ tumor-infiltrating lymphocytes (TILs) [139]. Better survival was linked to larger densities of proliferating CD8+ T cells and a higher ratio of CD8+ to CD4+ cells in tumor infiltrates [140].

Many clinical trials were performed and are still underway involving ICIs in adult GBM patients. The results of some trials revealed evidence of tumor immune microenvironment changes, such as increased immune cell infiltration, enhanced expression of chemokine transcripts, and expanded T-cell clonal diversity. These results implied that antibody-based treatments can produce immunomodulatory effects for tumors in the brain [141]. In most of the trials, the administration of the drugs was intravenous, raising doubts about whether suitable BBB penetration was achieved or whether local administration would have been better for biological activity.

Some of the clinical trials had good preclinical results when testing the efficacy of the anti-PD1 antibody nivolumab for treating GBM. One of the studies was CheckMate 143 [142], which evaluated the effects of nivolumab alone or combined with ipilimumab, an anti-CTLA-4 monoclonal antibody, in recurrent GBM. Phase 1 of the study proved that the toxicity profile was consistent with other cancers, with no new safety signals being identified. No evidence of clinically significant neurotoxicity was found, discarding the concerns existing due to the anatomical location of the tumor. The combination of nivolumab and ipilimumab was more toxic than nivolumab alone, being responsible for more frequent and severe immune-related adverse events. The tumors of the patients registered in the study had high PD-L1 expression (68%), there were some radiographic responses, and some of the patients displayed increased immune cell infiltrates on tissue biopsy [143]. Phase 3 of CheckMate 143 randomized recurrent glioblastoma patients into receiving nivolumab or anti-VEGF therapy bevacizumab [142]. The results showed that the OS was not improved in the nivolumab group, with no evidence of improved efficacy in PD-L1-expressing tumors. The same trial enrolled 136 patients with newly diagnosed glioblastoma. This group investigated the use of nivolumab with radiotherapy and temozolomide. In various cohorts of MGMT unmethylated patients, the temozolomide was removed due to a lack of efficacy in this phenotype. Results demonstrated that it is possible to use nivolumab and radiotherapy (RT), with or without temozolomide (TMZ). Higher toxicity was reported when both nivolumab and temozolomide were given. It was also reported a lower incidence of lymphopenia in the cohorts where temozolomide was omitted, with survival results depending on the MGMT methylation status. The median OS varied from 33.38 months in patients with methylated MGMT promoter treated with nivolumab + RT + TMZ to 16.49 months in the MGMT unmethylated group treated with nivolumab + RT + TMZ and 14.41 months in the MGMT unmethylated group treated with nivolumab + RT [144]. In other studies, CheckMate 498 [145] and CheckMate 548 [146,147] tested the efficiency of nivolumab together with radiation in MGMT-methylated and unmethylated newly diagnosed GBM patients. Checkmate 498 focused on unmethylated MGMT, trying to determine if nivolumab could be a potential replacement for temozolomide in the chemoresistant population [144]. Results showed no improvement in OS, this combination of therapies being inferior to chemotherapy. CheckMate 548, a randomized phase 3 study, tested the addition of nivolumab to standard chemoradiotherapy in MGMT methylated GBM, showing no improvement in OS [147]. No significant improvement in patient survival was observed in either of the three trials compared to standard treatment, and no correlation was found between PD-L1 expression and efficacy.

Pembrolizumab is another anti-PD-1 antibody that has been tested in glioblastoma. The use of pembrolizumab, either as monotherapy or in combination with bevacizumab, had very limited clinical benefit for recurrent GBM in various clinical trials [148,149,150,151]. The unfavorable results of the combination of pembrolizumab and bevacizumab discouraged the initial enthusiasm for the combination of ICIs and bevacizumab, which could have made possible the use of fewer corticosteroids, less synergistic effects, and the decrease of lymphocyte trafficking and cytokine release. One of the studies investigated the combination of pembrolizumab with hypofractionated stereotactic re-irradiation and bevacizumab for recurrent glioblastoma and anaplastic astrocytoma. The combination was advantageous, with a median PFS of 8 months and OS of 13.5 months in bevacizumab-naïve patients. Neoadjuvant treatment with anti-PD-1 showed good outcomes in selected recurrent GBM patients during window-of-opportunity trials [141,152]. Two small studies investigated the advantages of using pembrolizumab in recurrent glioblastoma prior to surgery as “neoadjuvant therapy” [141,152]. The analysis of post-treatment specimens revealed an increase in T-cell infiltration and antigen-reactive clonal expansion in the TME. One of the studies [152] focused on the potential clinical benefit of “neoadjuvant” usage. Following tumor removal, an amplified priming effect provides an optimal setting for ICI usage. Thirty-five patients with recurrent, surgically resectable GBM were randomized into two groups, either receiving neoadjuvant pembrolizumab with continuous administration following surgery (the neoadjuvant arm) or receiving only post-surgery pembrolizumab (adjuvant arm). The results showed a median OS of 13.7 months in the neoadjuvant arm compared to only 7.5 months in the adjuvant arm. It was also revealed that neoadjuvant PD-1 blockade was linked to the upregulation of T cell and interferon γ-related gene expression, the downregulation of cell-cycle related genes, enhanced clonal expression of T cells, focal induction of PD-L1 in the tumor microenvironment, decreased PD-1 expression on peripheral blood T cells, and decreased monocytic population, all of which are indicators of an improved immunological response. Another single-arm trial of neoadjuvant pembrolizumab found an OS of 20 months [150], even if the tumor sample analysis found a lack of immune activation markers, with a great amount of CD68+ macrophages. Because of the small number of patients included in these studies, it is impossible to draw any conclusions regarding the clinical benefits of neoadjuvant ICIs, with new studies in this area being necessary.

Atezolizumab is an anti-PD-L1 antibody that, in a small phase 1 trial, was found to be safe, with no real results regarding its efficacy being found because of the heavily pre-treated population enrolled in the study [153]. Other trials are still awaiting the publication of results.

Durvalumab is a human IgG1 monoclonal Ab against PD-L1. A phase 2 multi-center study explored the combination of durvalumab with standard radiotherapy in patients with unmethylated, newly diagnosed GBM. This combination was found to have favorable tolerability and may be efficient, with one patient achieving an OS of 86 weeks [154]. This can be explained by the fact that anti-PD1 and PD-L1 therapies may be potentiated by radiation due to the release of tumor antigens caused by induced cell death.

Ipilimumab is an anti-CTLA4 antibody approved by the FDA for treating other types of cancer. There is no clinical data regarding the use of ipilimumab as a single therapy for GBM due to the capacity of GBM to rapidly adapt to ICI therapy by increasing the expression of alternative checkpoints [155]. That is why the studies were focused on combinations of ipilimumab with other agents such as anti-PD1 blocking antibodies (NCT02311920, NCT04606316, NCT03233152, NCT04817254, NCT04145115, NCT043396860), VEGF inhibitors [156], tumor-treating fields (NCT03430791), TMZ, and radiotherapy (NCT03367715). Combining ipilimumab and nivolumab increased the immune toxicity compared to Nivolumab alone [157].

LAG-3 (lymphocyte activation gene 3), TIM-3 (T-cell immunoglobulin and mucin 3), TIGIT (T-cell immunoreceptor with Ig and ITIM domains), and IDO1 are the new targets being investigation in GBM [158]. These checkpoints are expressed on various immune cells, including T cells and NK cells, their obstruction being able to boost immune response against cancer cells. There are various clinical studies that investigate the LAG-3 blockade (relatlimab) as a single agent or combined with anti-PD-1 therapy in patients with newly diagnosed or recurrent GBM [159,160]. Another clinical trial is testing the inhibition of TIM-3 (sabatolimab) and PD-1 (spartalizumab) together with stereotactic radiosurgery in recurrent GBM (NCT03961971) and IDO in combination with radiotherapy or TMZ [161,162]. The combination of domvanalimab (targeting TIGIT) with Zimberelimab (targeting PD-1) has been shown to enhance T-cell responses in preclinical models of GBM, resulting in reduced tumor growth and improved survival [163]. An exploratory retrospective study [164] tried to find the optimal candidates for anti-PD-1 therapy. Enrichment of PTEN mutation was associated with immunosuppressive expression signatures in tumors that did not respond to nivolumab or pembrolizumab. In contrast, an enrichment of MAPK pathway mutations (PTPN11, BRAF) was found in responders. The latter was also associated with branched patterns of evolution from eliminating neoepitopes and with differences in T-cell clonal diversity and TEM profiles. These results are limited by the retrospective nature of the study.

The poor results of ICI treatments in GBM can be associated with various factors, such as the low mutational burden of GBM, high tumor heterogeneity, limited T-cell infiltration, intratumoral downregulation of MHC-I/MHC-II molecules, and the low drug penetration across the blood-brain barrier [100,165,166]. Some researchers are trying to combine laser interstitial thermal therapy (LITT) with ICIs, with promising benefits for recurrent GBM patients, LITT being able to ablate tumors and enhance drug penetration through the BBB breakdown [167,168,169].

In GBM, the use of ICIs has shown minimal clinical benefit, with most GBM patients not reacting to ICI therapy [138], whether applied individually or in combination (Table 1).

## 4. Combination Therapy

Because of the impossibility of treating a complex and heterogeneous tumor like GBM with a single treatment, researchers are now studying combination therapies, mixing immunotherapeutics with classical treatments and different immune-based approaches. Combination of ICIs and CAR-T-cell therapies, vaccination approaches, and oncolytic viruses such as AdVs (adenovirus) and PVSRIPO (a non-pathogenic poliovirus/rhinovirus chimeric virus with antineoplastic activity [170]) are being tested. It was reported that combining a specific expressing IL-12 oncolytic herpes simplex virus (oHSV) with anti-PD-1 and anti-CTLA-4 checkpoint inhibitors could be curative [171]. This complex of therapies could be effective because of the participation of CD4+ and CD8+ T cells, together with macrophages, building an intricate successful interaction.

A phase 1/2 trial, which combined the use of pembrolizumab with oncolytic virotherapy in recurrent glioblastoma, was completed with an objective response rate of 10.4% and OS rate at 12 months of 52.7% after intratumoral administration of virus DNX-2401, followed by intravenous administration of pembrolizumab. Three out of 49 patients were alive at 60 months [172].

The combination of radiotherapy, which is basically immunogenic, being able to reprogram an immunosuppressive TME [173], with various forms of immunotherapy is an active area of research. Radiation was linked to dynamic alterations in tumor-associated macrophages and microglia in glioma, the implications of these mechanisms being still unclear [174]. Understanding how radiation influences the immune response is especially important for the improvement of outcomes and the development of effective combination therapies.

Standard chemotherapy induces immunosuppression and lymphopenia in GBM patients, being an important obstacle to GBM-immune-based treatments. It is important to find new standard therapies to increase the success of immunotherapies [175]. Targeting a single axis, like a single antigen or immune checkpoint molecule, seems insufficient.

## 5. Potential Biomarkers to Evaluate the Effect of ICIs in the Treatment of Cancer

Immune checkpoint inhibitors have changed the way many types of cancer are clinically treated, and many predictive biomarkers are already used for immunogenic tumor types. There is an urgent need for biomarkers that can identify glioma patients who will have a good response to ICIs. One of the biomarkers used for other types of cancer is the expression of PD-L1 on tumor and immune cells. Other options may be represented by T- cell infiltration, certain gene mutations, neoantigens expression, DNA deficient mismatch repair (dMMR), high levels of microsatellite instability (MSI-H) across the genome, and high tumor mutational burden (TMB) [176,177,178,179]. These biomarkers are not very frequent in glioblastoma, and they only appear in small subsets of tumors, explaining to some extent why the majority of glioblastomas are unresponsive to treatment [149,166,180,181]. PD-L1 is present in nearly 90% of GBM cells [182], its expression being heterogeneous within tumors and in peripheral immune cells of glioma patients [181,183]. Studies have shown that the expression of PD-L1 is lower in glioma-infiltrating MDMs than in brain metastases infiltrating MDMs [184], being a possible explanation for the lack of relationship between PD-L1 expression and survival in clinical trials [145,146,149].

In other cancers, in order to assess the accuracy of predictive biomarkers, a multicomponent panel that includes clinical, genomic, and transcriptomic variables was developed [185]. Hence, it is possible that integrating multiple biomarkers will also be needed to identify patients who will benefit from using ICIs in glioblastoma.

Although one meta-analysis failed to find evidence of improved response in patients with high TMB [186], and another clinical study implies that recurrent tumors with very low TMB are more responsive to immunotherapy [187], at present, the only approved indication for the use of ICIs in glioblastoma is for recurrent tumors with a high TMB, more than 10 mutations per megabase [165]. TMZ-hypermutated GBM patients do not show an unusual T-cell infiltration and did not experience any change in survival after using ICIs [188]. TMB induces clonal (present in all tumor cells) and subclonal (present in only a subset of tumor cells) neoantigens. When the use of pembrolizumab in the treatment of non-small cell lung cancer was studied, it was demonstrated that only clonal neoantigens were recognized by T cells, resulting in clonal TMB being an important marker of response to ICIs, compared to subclonal mutations [189,190]. Evidence is pointing out the importance of clonality and quality, rather than quantity, of neoantigens, for immunotherapy efficacy [191].

It was recently discovered that defects in DNA replication stress response may predict clinical outcomes of ICI use in GBM patients [192]. In studies that evaluated PD-1 blockade in recurrent GBM, it was observed that better outcomes were associated with a gene expression signature that predicts functional defects in the DNA replication stress response, implying that the use of PD-1 checkpoint inhibitors may be useful in patients identified by their DNA damage and replication stress molecular status [152,164].

Two studies designed to identify molecular characteristics of recurrent GBM patients treated with adjuvant PD-1 blockade that were responsive to treatment identified the enrichment of BRAF or PTPN11 activating mutations in approximately 30% of the tumors [164,193]. These mutations appear with a frequency of only 2–3% among GBMs [36]. BRAF/PTPN11 mutations activate the signaling of MAPK (mitogen-activated protein kinase) pathway. Phosphorylated extracellular signal-regulated kinase 1/2 (p-ERK), a member of the MAPK family, was found in tumors of patients with longer survival after using PD-1 inhibitors as adjuvant treatment in recurrent glioblastoma [193]. In patients not treated with immunotherapy, p-ERK levels were not linked to survival.

## 6. Neurotoxicity Profile of Immune Checkpoint Inhibitors

The use of antibody therapies can cause adverse effects on normal tissues, affecting treatment efficacy. These immune-related adverse effects can include thyroiditis, colitis, rashes, hepatitis, pneumonitis, and neurologic syndromes. These appear due to the overactivation of the peripheral immune system, failure of self-tolerance, and activation from viral or co-administered drug antigens [194,195]. Neurological adverse effects were described by multiple case reports, and researchers found that up to 4.2% of cancer patients treated with anti-PD-1 drugs had some neurological dysfunctions, most of them emerging within 3 months of starting the therapy [196]. Combination therapies raise the risk by up to 12% [197]. Both the central and peripheral nervous systems are affected by the immune-related adverse effects, with syndromes that include seizures, peripheral neuropathy, headaches, diffuse encephalopathy, demyelinating diseases, CNS vasculitis, aseptic meningitis, and neuromuscular receptor dysfunction [198,199,200].

The subtle balance between efficacy and toxicity remains a crucial component of immunotherapy, and further research is needed to control the immune-related adverse effects.

## 7. Conclusions and Future Directions

The poor prognosis of GBM patients demands new treatments to improve both quality of life and overall survival. Although immunotherapeutic treatments offer remarkable results in treating other solid tumors, they have limited results in GBM. No significant outcomes were found after using anti-GBM immunotherapeutics in phase 3 clinical trials, whether tested individually or in combination with standard treatments. This failure highlights the need to better understand the glioblastoma biology, including local tumor microenvironment immunosuppression and systemic T-cell dysfunction. There are mechanisms related to the tumor’s location, growing in an immune-privileged site, and there are also underlying mechanisms of resistance that are probably shared with other solid tumors that are not responsive to ICIs. The tumor physical microenvironment (TPME) contributes to cancer chemoresistance in both mechanical (ECM physical features) and mechanobiological approaches (ECM-responsive signaling pathways). After chemotherapy exposure, the TME undergoes significant changes that are able to regulate TPME through ECM remodeling. Recently, new information emerged regarding how radiotherapy influences the biophysical cues (i.e., microarchitecture, stiffness, solid stress, and interstitial fluid pressure) in TME. It is important to explore the interaction between the immune system and ECM changes caused by chemotherapeutic exposure and radiotherapy as well, in order to better understand the implications and develop better treatment options for GBM [201,202] Immunotherapy can be combined with radiotherapy, chemotherapy, and nano-medicine to achieve TME normalization.

So far, the experience of using ICIs to treat GBM has offered disappointing outcomes, but this approach remains of high interest in neuro-oncology. Further research is needed to find new ways of selecting the best candidates and to improve the understanding of response and resistance to ICI. Using biomarkers would significantly help in conceiving a personalized treatment approach for each patient.

The fact that only T cells previously activated in the periphery can enter the CNS adds to the challenge represented by the blood-brain barrier, which can limit leukocyte trafficking. The CNS T-cell population is characterized by tissue-resident memory, being also enriched for viral specificities, allowing only limited antigen presentation possible in CNS tumors [126]. The deficient lymphatic drainage of the CNS has been found to play an important part in the poor T-cell activation observed in glioblastoma models [201].

To conclude, the use of antibody-based therapies offers promising new potential in neuro-oncology. The success of ICI use will depend on defeating the two main obstacles: the BBB and the immunosuppressive TME. Scientists are trying to find new and better ways to deliver the drugs into the CNS with the help of transcytosis, focused ultrasound, and nanoparticles.

In many different tumor types, the use of ICIs has improved life expectancy, changing how cancer is treated. An understanding of the processes driving resistance will help in the development of better next-generation immunotherapies. Nowadays, glioblastoma remains incurable, resulting in an urgent need to develop new approaches for its management.

## Figures and Tables

**Table 1:** List of ICIs being currently studied. (New D GBM—newly diagnosed glioblastoma).

Immune Checkpoint Inhibitor	Molecular Target	Intervention	Conditions	Author
Nivolumab	Anti-PD1	Monotherapy +bevacizumab +/−ipilimumab +radiotherapy +/− TMZ	Recurrent GBM NewD GBM	Reardon DA, et al. [142] Omuro A, et al. [143] Omuro A, et al. [144] Omuro A, et al. [145] Lim M, et al. [146], Weller M, et al. [147]
Pembrolizumab	Anti-PD1	Monotherapy +bevacizumab +hypofractioned stereotactic re-irradiation + bevacizumab Neoadjuvant therapy +oncolytic virotherapy	Recurrent GBM Recurrent GBM + anaplastic astrocytoma Recurrent GBM Recurrent GBM	Reardon DA, et al. [148], Nayak L, et al. [149], Lombardi G, et al. [151] Schalper KA, et al. [141], Cloughesy TF, et al., [152], De Groot J, et al. [150] Gromeier M, et al. [170], Nassiri F, et al. [171]
Atezolizumab	Anti-PD-L1	Monotherapy	Recurrent GBM	Lukas RV, et al. [153]
Durvalumab	Anti-PD-L1	+radiotherapy	Unmethylated NewD GBM	Reardon DA, et al. [154]
Ipilimumab	Anti-CTLA4	Monotherapy + bevacizumab +TTF +TMZ and radiotherapy	NewD GBM	Carter T, et al. [156]
Relatlimab	Anti—LAG3	Monotherapy +anti-PD-1	NewD GMB, Recurrent GBM	Lim M, et al. [159], Lynes J, et al. [160]
Sabatolimab+ Spartalizumab	Anti—TIM3 Anti-PD1	+stereotactic radiosurgery	Recurrent GBM	
Indoximod	Anti—IDO1	+radiotherapy/TMZ +nivolumab	Recurrent GBM	Zakharia Y, et al. [161], Lukas R, et al. [162]
Domvanalimab	Anti-TIGIT	+zimberelimab (anti PD-1)	Preclinical models of GBM	Hung AL, et al. [163]

## Data Availability

Not applicable.
